# A metabolome-wide association study in the general population reveals decreased levels of serum laurylcarnitine in people with depression

**DOI:** 10.1038/s41380-021-01176-0

**Published:** 2021-06-04

**Authors:** Helena U. Zacharias, Johannes Hertel, Hamimatunnisa Johar, Maik Pietzner, Karoline Lukaschek, Seryan Atasoy, Sonja Kunze, Henry Völzke, Matthias Nauck, Nele Friedrich, Gabi Kastenmüller, Hans J. Grabe, Christian Gieger, Jan Krumsiek, Karl-Heinz Ladwig

**Affiliations:** 1 Department of Psychiatry and Psychotherapy, University Medicine Greifswald, Greifswald, Germany; 2 Department of Internal Medicine I, University Medical Center Schleswig-Holstein, Kiel, Germany; 3 Institute of Clinical Molecular Biology, Kiel University, University Medical Center Schleswig-Holstein, Kiel, Germany; 4 School of Medicine, National University of Ireland, Galway, Ireland; 5 Institute of Epidemiology, Helmholtz Zentrum München, Neuherberg, Germany; 6 Department of Psychosomatic Medicine and Psychotherapy, University of Gießen and Marburg, Gießen, Germany; 7 Institute of Clinical Chemistry and Laboratory Medicine, University Medicine Greifswald, Greifswald, Germany; 8 Computational Medicine, Berlin Institute of Health (BIH), Charité—Universitätsmedizin Berlin, Berlin, Germany; 9 Institute of General Practice and Family Medicine, University Hospital of the Ludwig-Maximilians University of Munich, Munich, Germany; 10 Department of Psychosomatic Medicine and Psychotherapy, Klinikum rechts der Isar, Technische Universität München, Munich, Germany; 11 Research Unit of Molecular Epidemiology, Helmholtz Zentrum München, Neuherberg, Germany; 12 Institute of Community Medicine, University Medicine Greifswald, Greifswald, Germany; 13 German Center for Cardiovascular Research (DZHK e.V.), partner site Greifswald, Greifswald, Germany; 14 Institute of Computational Biology, Helmholtz Zentrum München, Neuherberg, Germany; 15 German Centre for Neurodegenerative Diseases (DZNE), Site Rostock/Greifswald, Germany; 16 German Center for Diabetes Research (DZD e.V.), Neuherberg, Germany; 17 Department of Physiology and Biophysics, Weill Cornell Medicine, Institute for Computational Biomedicine, Englander Institute for Precision Medicine, New York, NY USA

**Keywords:** Psychology, Molecular biology

## Abstract

Depression constitutes a leading cause of disability worldwide. Despite extensive research on its interaction with psychobiological factors, associated pathways are far from being elucidated. Metabolomics, assessing the final products of complex biochemical reactions, has emerged as a valuable tool for exploring molecular pathways. We conducted a metabolome-wide association analysis to investigate the link between the serum metabolome and depressed mood (DM) in 1411 participants of the KORA (Cooperative Health Research in the Augsburg Region) F4 study (discovery cohort). Serum metabolomics data comprised 353 unique metabolites measured by Metabolon. We identified 72 (5.1%) KORA participants with DM. Linear regression tests were conducted modeling each metabolite value by DM status, adjusted for age, sex, body-mass index, antihypertensive, cardiovascular, antidiabetic, and thyroid gland hormone drugs, corticoids and antidepressants. Sensitivity analyses were performed in subcohorts stratified for sex, suicidal ideation, and use of antidepressants. We replicated our results in an independent sample of 968 participants of the SHIP-Trend (Study of Health in Pomerania) study including 52 (5.4%) individuals with DM (replication cohort). We found significantly lower laurylcarnitine levels in KORA F4 participants with DM after multiple testing correction according to Benjamini/Hochberg. This finding was replicated in the independent SHIP-Trend study. Laurylcarnitine remained significantly associated (*p* value < 0.05) with depression in samples stratified for sex, suicidal ideation, and antidepressant medication. Decreased blood laurylcarnitine levels in depressed individuals may point to impaired fatty acid oxidation and/or mitochondrial function in depressive disorders, possibly representing a novel therapeutic target.

## Introduction

Depression and related conditions are among the most common psychiatric disorders worldwide [1]. Characterized by profound sustained negative affectivity and reduced drive and energy [2] as its key elements, depression severely limits daily functioning and leads to substantial impairments in quality of life of affected subjects [3]. Depression results from a complex interaction of social, psychological, and biological factors. Adverse psychological conditions, negative life experiences and trauma, particularly in childhood [4] and often amplified by social isolation or loneliness, contribute to depression onset. Thus, depression is linked to a state of chronic psychosocial stress likely to affect multiple psychobiological systems with impairments of the HPA axis [5] and activation of subclinical chronic inflammation in the absence of inhibitory feedback [6] among the most prominent central pathways between the adverse mental disease condition and its physiological underpinning. However, the predictive values of these single domains remain modest, their cross-talk is poorly understood and translation into evidence based treatment regimens scarce. A deeper understanding of signaling molecules is still in its infancy [7–9].

Metabolomics has recently emerged as a particularly valuable tool for exploring molecular pathways in psychiatry because it captures the final products of complex interactions among numerous proteins, signaling cascades, and cellular environments [10–13]. Metabolomics research can follow a targeted or untargeted approach. The first strategy focusses on the exact determination and statistical analysis of a preselected set of metabolites, whereas the latter aims at the maximization of metabolite coverage and considers all detectable molecules in the subsequent analysis. The latter approach facilitates the detection of previously unknown biomarkers, enabling a more holistic and unbiased view on associations between the human metabolism and external stimuli. The task of a metabolome-wide association analysis is to discover such metabolic biomarkers, which constitute a “metabolic fingerprint” or “metabolic signature” of the investigated phenotype.

To date, several studies revealed distinct metabolic fingerprints of depression-related phenotypes, including major depressive disorder (MDD) [8, 14–22], severity of depression [23], melancholic depression [7, 15], depression recovery [11], and pharmacologically induced immune-related depression [24]. Most of these studies, however, only employed a targeted metabolomics approach [11, 14, 17, 22], and/or restricted their analyses to specific patient subcohorts [11, 14, 21, 23, 24], with low sample sizes and lacking external replication.

Thus, the major aim of the present investigation was to gain a deeper understanding of the complex network of metabolic factors associated with depression. We performed a comparative untargeted analysis with thorough adjustment for possible metabolic and medication confounders of an extended array of 353 unique serum metabolites in a general population-based study sample of KORA (Cooperative Health Research in the Augsburg Region) F4 comprising, in total, 1411 individuals. Secondly, we aimed to validate our metabolic findings in an independent, large-scale general population sample consisting of 968 participants of the SHIP-Trend (Study of Health in Pomerania) study. Additional sensitivity analyses were carried out in subcohorts stratified according to sex, suicidal ideation and current use of antidepressants to assess potential differences in metabolite associations. Both KORA and SHIP-Trend offer high-quality biomedical and metabolomics data assessed by standardized protocols, enabling the detection of novel metabolic biomarkers of depression in the general population.

## Materials and methods

### Study cohorts

Our discovery cohort consisted of 1411 participants of the KORA (Cooperative Health Research in the Augsburg Region) F4 study (2006–2008), a follow-up study of the 4th KORA survey (S4) carried out in 1999–2001. The KORA S4 is a cross-sectional, population-based study, recruiting participants from the Augsburg region, and two adjacent counties in southern Germany [25]. The F4 study examined 3080 subjects aged 32–81 years at follow-up on average 7 years after the baseline examination. Out of the F4 study, participants aged 32–77 years were randomly chosen for metabolomic characterization and genome-wide association studies with metabolic traits [26, 27]. Each study participant provided informed consent and all study protocols were approved by the ethics committee of the Bavarian Medical Association in Munich, Germany.

Our replication cohort comprised 968 study participants without self-reported diabetes of the SHIP-Trend (Study of Health in Pomerania) study [28], for whom mass-spectrometry (MS)-based plasma metabolomics as well as 9-item Patient Health Questionnaire (PHQ-9) data were available. SHIP-Trend is a population-based study, recruiting study participants in West Pomerania, and baseline examinations were carried out in 2008–2012. For each study participant, written informed consent is available and the study was approved by the Institutional Ethics and Scientific Review Committee of the University Medicine Greifswald.

### Psychological characteristics

The PHQ-9 was administered in personal interviews to assess depressive symptoms in both cohorts. It captures depressive symptoms on a scale of 0–27. Three categories, “no”, “other”, and “major depressive symptoms” were defined for the KORA F4 discovery cohort [29, 30]. Details about depression and suicidal categorization, the intake of antidepressants and assessment of cardiometabolic risk factors are provided in the Supplementary Information.

### Metabolomics profiling

From each KORA study participant, blood samples were collected between 8 and 11 a.m. after an overnight fast of at least 8 h and subsequently stored at −80 °C as described previously [26, 31]. Metabolomics profiling of serum specimens was conducted by Metabolon (Research Triangle Park, NC, USA) with two separate ultra-high performance liquid chromatography/tandem MS (UHPLC/MS/MS; Thermo Scientific, USA) injections and one gas chromatography/MS (GC/MS) injection for each sample [32].

From each SHIP-Trend study participant, fasting blood samples were collected between 7 and 13 a.m. as described previously [33]. A targeted metabolic profiling approach for the plasma specimens was carried out using the AbsoluteIDQ p180 Kit (BIOCRATES LifeSciences AG, Innsbruck, Austria) as detailed in [33]. Details regarding data preprocessing are given in the Supplementary Information.

### Statistical analysis

For descriptive analyses, differences in frequencies and means were tested employing Chi-squared or *t* tests, with a significance level of 0.05.

To identify metabolites associated with depression in KORA F4, we employed a linear regression test modeling each metabolite value by the categorical depression status, comparing either “no” vs. “other/major depressive symptoms” combined or “no” vs. “other” or vs. “major depressive symptoms” separately. Data normality was confirmed by visual inspection of residual diagnostic plots. Age, sex, body-mass index (BMI), and the intake of antihypertensive, cardiovascular, antidiabetic, and thyroid gland hormone drugs, as well as corticoids, and antidepressants were included as confounders (Supplementary Table S1). To account for multiple testing, two-sided *p* values were adjusted according to the method of Benjamini/Hochberg (B/H) [34] to control the false discovery rate (FDR). An association was considered to be statistically significant, if its corresponding B/H-adjusted *p* value was below 0.05, corresponding to an FDR of 5%.

In SHIP-Trend, we employed linear regression models to assess the association between laurylcarnitine levels (per 1-unit increases) and the sum of PHQ-9 scores, adjusted for age, sex, BMI, and the intake of antihypertensive drugs, antithrombotic agents, HMG CoA reductase inhibitors, nonsteroid anti-inflammatory and antirheumatic drugs as well as antidepressants (Supplementary Table S1). For these replication analyses, only one-sided *p* values were considered.

Sensitivity analyses in KORA F4 were performed by stratifying study participants into men/women, people with/without suicidal ideation, and intake/no intake of antidepressants, respectively. Here, in comparison to the linear regression approach, all assessed antidepressants were considered, i.e., tricyclic antidepressants, selective serotonin reuptake inhibitors, mirtazapine antidepressants, lithium containing antidepressants, and other antidepressants.

Statistical analyses were conducted with *R* version 3.4.3 [35].

## Results

### Baseline characteristics of subjects with depression

In total, 1411 study participants of the KORA F4 (48.5% men, 51.5% women), with a mean age of 58.6 ± 7.49 years and BMI of 27.98 ± 4.79 kg/m^2^ were included in this study among them 72 (5.1%) participants suffering from depression. Depressed subjects had a significantly higher BMI, a higher prevalence of type-2 diabetes mellitus, and were more likely to report suicidal ideation in comparison to subjects without depression (all *p* values < 0.05). They received significantly more ACE inhibitors, antidiabetic medication, tricyclic antidepressants, selective serotonin reuptake inhibitors, mirtazapine antidepressants, as well as lithium containing antidepressants (Table 1).

**Table 1:** Baseline characteristics of KORA F4 study participants stratified according to depression status (*n* = 1411).

	No depressive symptoms	Other/major depressive symptoms	Other depressive symptoms	Major depressive symptoms	*p* value no vs. other/major depressive symptoms	*p* value no vs. other depressive symptoms	*p* value no vs. major depressive symptoms
*n* (%)	1339 (94.9%)	72 (5.1%)	51 (3.6%)	21 (1.5%)	–	–	–
male (%)	652 (48.7%)	33 (45.8%)	25 (49.0%)	8 (38.1%)	0.725^a^	1^a^	0.457^a^
BMI [kg/m^2^]	27.91 ± 4.74	29.29 ± 5.53	29.30 ± 5.66	29.26 ± 5.33	0.041^b^	0.089^b^	0.263^b^
Type-2 diabetes mellitus (%)	107 (8.0%)	12 (16.7%)	7 (13.7%)	5 (23.8%)	0.018^a^	0.228^a^	0.027^a^
Age [years]	58.53 ± 7.49	59.15 ± 7.12	58.35 ± 6.88	61.10 ± 7.48	0.471^b^	0.861^b^	0.133^b^
Hypertension^c^ (%)	543 (40.6%)	33 (45.8%)	23 (45.1%)	10 (47.6%)	0.644^a^	0.784^a^	0.798^a^
Suicidal ideation (%)	38 (2.8%)	20 (27.8%)	8 (15.7%)	12 (57.1%)	<2.2 e^−16a^	<2.2 e^−16a^	3.6 e^−6a^
Intake of antihypertensive drugs (%)	418 (31.2%)	30 (41.7%)	21 (41.2%)	9 (42.9%)	0.084^a^	0.178^a^	0.366^a^
Intake of antihypertensive medication with beta-blockers (%)	255 (19.0%)	20 (27.8%)	12 (23.5%)	8 (38.1%)	0.095^a^	0.537^a^	0.056^a^
Intake of ACE inhibitors (%)	156 (11.7%)	17 (23.6%)	16 (31.4%)	1 (4.8%)	0.005^a^	6.9e^−5a^	0.525^a^
Intake of angiotensin-antagonists (%)	117 (8.7%)	7 (9.7%)	5 (9.8%)	2 (9.5%)	0.941^a^	0.990^a^	1^a^
Intake of lipid-lowering medication (%)	229 (17.1%)	19 (26.4%)	14 (27.5%)	5 (23.8%)	0.063^a^	0.085^a^	0.605^a^
Intake of statins (%)	181 (13.5%)	8 (11.1)	7 (13.7%)	1 (4.8%)	0.684^a^	1^a^	0.397^a^
Intake of antidiabetic medication (%)	58 (4.3%)	10 (13.9%)	7 (13.7%)	3 (14.3%)	0.0007^a^	0.0054^a^	0.098^a^
Intake of thyroid medication (%)	202 (15.1%)	7 (9.7%)	5 (9.8%)	2 (9.5%)	0.281^a^	0.401^a^	0.689^a^
Intake of systemic corticoids (%)	20 (1.5%)	1 (1.4%)	1 (2.0%)	0 (0%)	1^a^	1^a^	1^a^
Intake of tricyclic antidepressants (%)	23 (1.7%)	8 (11.1%)	5 (9.8%)	3 (14.3%)	1.0 e^−^^6^^a^	0.0004^a^	0.0008^a^
Intake of selective serotonin reuptake inhibitors (%)	18 (1.3%)	4 (5.6%)	1 (2.0%)	3 (14.3%)	0.020^a^	1^a^	0.0001^a^
Intake of other antidepressive medication (%)	7 (0.5%)	1 (1.4%)	0 (0%)	1 (4.8%)	0.882^a^	1^a^	0.279^a^
Intake of mirtazapine antidepressants (%)	5 (0.4%)	2 (2.8%)	1 (2%)	1 (4.8%)	0.049^a^	0.543^a^	0.177^a^
Intake of lithium containing antidepressants (%)	3 (0.2%)	2 (2.8%)	2 (3.9%)	0 (0%)	0.011^a^	0.002^a^	1^a^

For continuous variables, values are expressed as mean ± standard deviation, for categorical variables, values are expressed as counts and frequencies.

*BMI* body-mass index.

^a^*P* values were calculated using Pearson’s Chi-squared test with Yates’ continuity correction.

^b^*P* values were calculated using a two-sided *t* test assuming unequal variance.

^c^For the diagnosis of hypertension, two values were missing.

From the Ship-Trend baseline cohort, 968 study participants (44% men, 56% women) were eligible for the replication analyses. In comparison to the KORA F4 discovery cohort sample, the SHIP-Trend study sample comprised significantly more women; they were significantly younger (mean age 50.02 ± 13.66 years), and had a lower BMI (27.36 ± 4.59 kg/m^2^). A total of 52 (5.4%) SHIP-Trend study participants were diagnosed with depression including 15 (1.6%) exhibiting major and 37 (3.8%) other depressive symptoms, respectively. Depressed SHIP-Trend study participants received significantly more nonsteroid anti-inflammatory and antirheumatic drugs, nonselective serotonin reuptake inhibitors, St.-John’s-wort, and other antidepressants in comparison to nondepressed subjects (Supplementary Table S2).

### Screening for metabolic depression markers in KORA F4

**Fig. 1:**
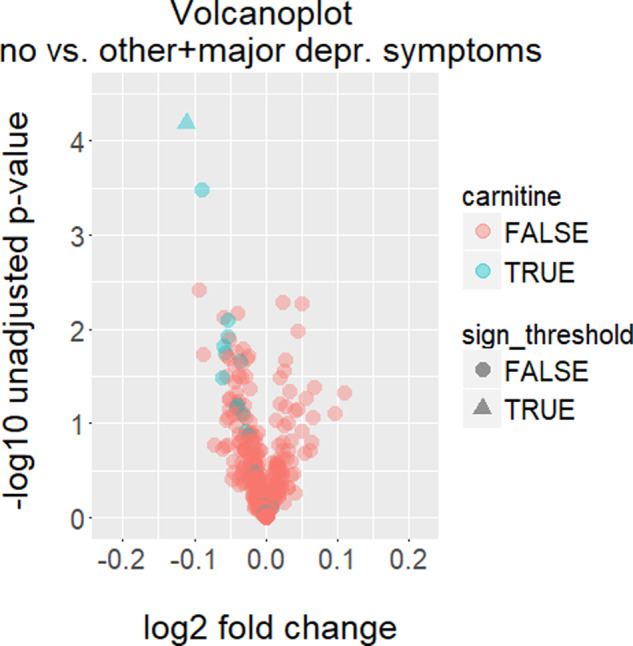
Volcanoplot illustrating the results of the screening for serum metabolic markers for other and major depression in KORA F4. Study participants without depressive symptoms were compared against study participants with other/major depressive symptoms. The *x*-axis shows the log_2_-fold changes, whereas the *y*-axis shows the −log_10_ unadjusted *p* values, respectively. Metabolites with Benjamini/Hochberg-adjusted two-sided *p* values below the significance threshold of 0.05 are represented as triangles, whereas all other metabolites are represented as dots. All metabolites identified as carnitines are plotted in blue, all other metabolites, including not yet identified metabolites, are plotted in red, respectively.

We tested 353 serum metabolites for a linear association with depressive symptoms in KORA F4, adjusted for age, gender, BMI, and the intake of antihypertensive, cardiovascular, antidiabetic, and thyroid gland hormone drugs, corticoids, as well as antidepressants (Table 2, Fig. 1, Supplementary Tables S3–5, and Fig. S1). Our metabolomics data covered a broad range of different metabolic classes including amino acids, carbohydrates, cofactors and vitamins, lipids, nucleotides, peptides, xenobiotics, as well as metabolites involved in energy metabolism. The linear regression test comparing study participants without depression versus participants with other/major depressive symptoms combined revealed one statistically significant metabolite identified as laurylcarnitine with a B/H-adjusted *p* value of 0.023 (Table 2). The same metabolite also obtained the lowest significant B/H-adjusted *p* value of 0.030 in the comparison no versus major depressive symptoms (Supplementary Table S5). For the comparison no versus other depressive symptoms, the smallest B/H-adjusted *p* value of 0.672 was obtained for an unknown metabolite (Metabolon identifier X12244; Supplementary Table S4). Laurylcarnitine was negatively associated with both other/major and major depressive symptoms, with relative differences (regression coefficients *β*) of −0.111 and −0.199, respectively (Table 2 and Supplementary Table S5). In fact, it was also negatively associated with other depressive symptoms (*β* = −0.076), although it only reached nominal significance with an unadjusted *p* value of 0.020 (B/H-adjusted *p* value 0.672) (Supplementary Table S4).

**Table 2:** Serum metabolites strongly (unadjusted *p* value < 0.01) associated with other/major depression in KORA F4.

Metabolite	Subpathway	Superpathway	Unadjusted *p* value	BH-adjusted *p* value	Relative difference (*β*)
Laurylcarnitine	Carnitine metabolism	Lipid	6.60e^−5^	0.023	−0.111
Decanoylcarnitine	Carnitine metabolism	Lipid	0.00033	0.057	−0.090
X- 12092			0.00385	0.350	−0.093
Phosphate	Oxidative phosphorylation	Energy	0.00518	0.350	0.023
X- 14056			0.00541	0.350	0.051
X- 11809			0.00683	0.350	−0.040
Homostachydrine*	Urea cycle; arginine-, proline-, metabolism	Amino acid	0.00747	0.350	−0.058
Palmitoylcarnitine	Carnitine metabolism	Lipid	0.00794	0.350	−0.054

The first column gives the names of the metabolites or their Metabolon identifier, if not yet identified, followed by the corresponding sub- and superpathways as provided by Metabolon Inc., the fourth and fifth columns list the unadjusted and adjusted *p* values according to Benjamini/Hochberg (BH), respectively, and the last column lists the relative differences (regression coefficients *β*). The regression analysis included the confounders age, sex, body-mass index (BMI), and the intake of antihypertensive, cardiovascular, antidiabetic, and thyroid gland hormone drugs, as well as corticoids, and antidepressants. No depressive symptoms and other/major depressive symptoms were recoded into 0 and 1, respectively. Thus, a positive *β* indicates an upregulation of the respective metabolite in the depression group. Supplementary Table S3 provides the complete results of the screening for metabolic depression markers in KORA F4.

Interestingly, the majority of carnitine metabolites was negatively associated with depression (Fig. 1 and Supplementary Fig. S1), although none of them except for laurylcarnitine reached the significance threshold. Supplementary Table S6 provides an overview of carnitine metabolites measured by the KORA F4 Metabolon approach. Acylcarnitines, nominally associated with other/major depressive symptoms, were decanoylcarnitine, palmitoylcarnitine, hexanoylcarnitine, cis-4-decenoyl carnitine, octanoylcarnitine, 2-methylbutyroylcarnitine, and 2-tetradecenoyl carnitine (Supplementary Table S3). The following acylcarnitines were nominally associated with major depression: palmitoylcarnitine, decanoylcarnitine, stearoylcarnitine, cis-4-decenoyl carnitine, 2-tetradecenoyl carnitine, and oleoylcarnitine (Supplementary Table S5). For the comparison of no vs. other depressive symptoms decanoylcarnitine, 2-methylbutyroylcarnitine, isobutyrylcarnitine, hexanoylcarnitine, and isovalerylcarnitine were nominally associated with depression (Supplementary Table S4). We observed strong correlations amongst the individual acylcarnitines (Supplementary Table S7).

### Sensitivity analysis for laurylcarnitine in KORA F4

**Fig. 2:**
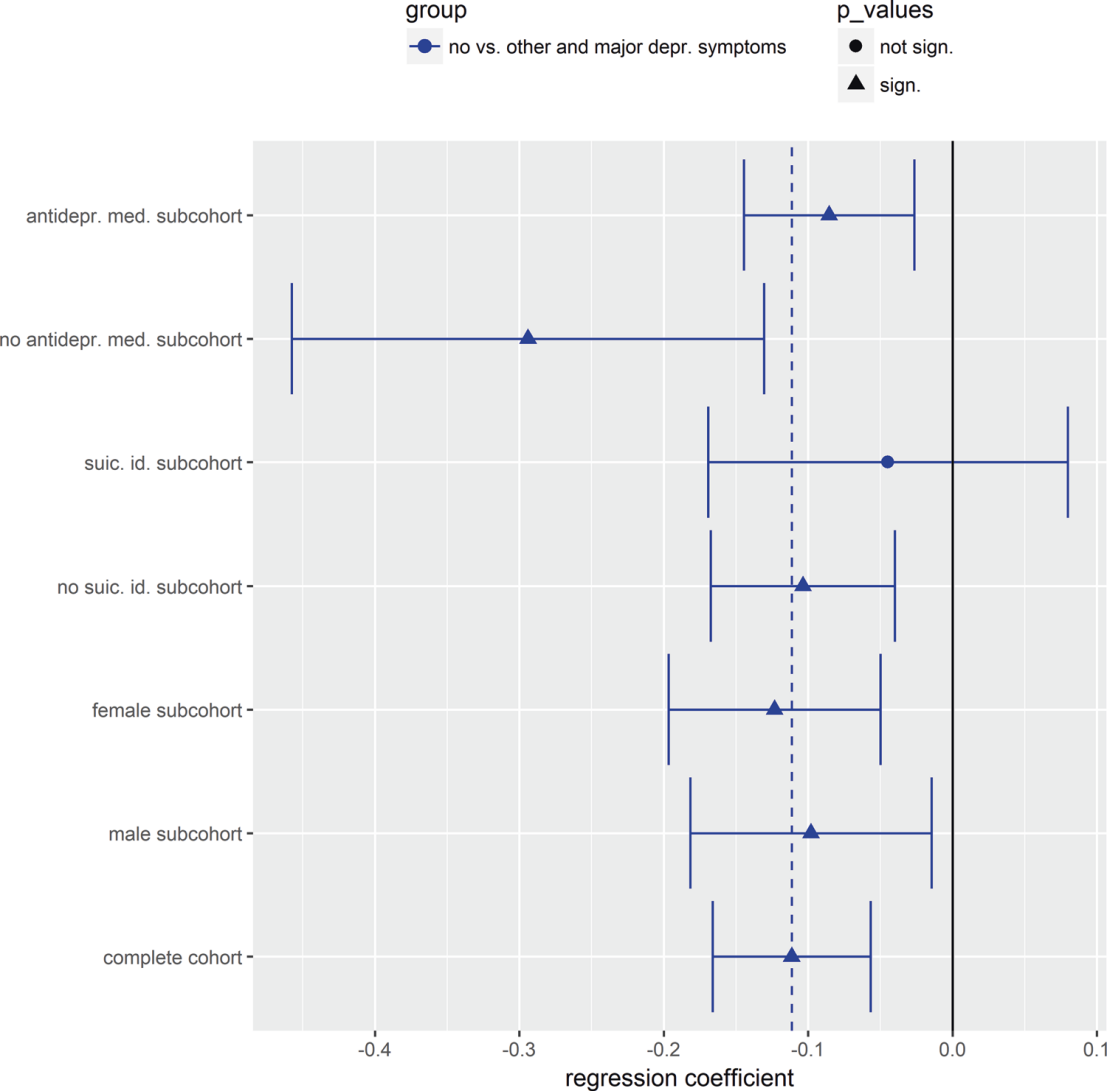
Estimated regression coefficients *β* (effect sizes) for the linear regression analyses between serum laurylcarnitine (outcome) and other/major depressive symptoms (predictor) stratified according to different subpopulations in KORA F4. The mean of the estimated coefficient is represented either as a triangle in the case of a significant association with laurylcarnitine (unadjusted two-sided *p* value < 0.05), or as a dot in the case of a nonsignificant association with laurylcarnitine. The whiskers correspond to the 95% confidence intervals. Regression coefficients for a comparison between no vs. other/major depressive symptoms are plotted in blue. Dashed vertical lines represent regression coefficients in the complete cohort and *β* = 0 is marked by a solid black line. antidepr. med. antidepressive medication, suic. id. suicidal ideation.

Next, we performed linear regression analyses to examine the association between depression and laurylcarnitine levels in KORA F4, stratified according to sex, history of suicidal ideation, and use of antidepressants considering the same confounders as above (Fig. 2 and Supplementary Fig. S2).

In men, laurylcarnitine was still nominally negatively associated with other/major (unadjusted *p* value 0.022, *β* = −0.098), and major depressive symptoms (unadjusted *p* value 0.017, *β* = −0.203), respectively. In women, laurylcarnitine was also significantly negatively associated with other/major (unadjusted *p* value 0.001, *β* = −0.123), and major depressive symptoms (unadjusted *p* value 0.001, *β* = −0.205), respectively.

In the analyses stratified by the presence of suicidal ideation, laurylcarnitine was significantly negatively associated with depression only in the subgroup without suicidal ideation with unadjusted *p* values for the comparison between subjects with no vs. other/major, no vs. other, or no vs. major depressive symptoms of 0.001 (*β* = −0.103), 0.040 (*β* = −0.073), and 0.001 (*β* = −0.253), respectively.

Following stratification according to antidepressant intake, laurylcarnitine remained significantly associated with major depressive symptoms in both subgroups not taking and taking antidepressants with unadjusted *p* values of 0.0037 (*β* = −0.324) and 0.014 (*β* = −0.145), respectively. Laurylcarnitine was also significantly associated with other depressive symptoms in the subgroup not taking antidepressants (unadjusted *p* value 0.027, *β* = −0.259). After combining patients with other and major depressive symptoms, we obtained significant *p* values of 0.0007 (*β* = −0.294) and 0.005 (*β* = −0.085) for participants treated or not treated with antidepressants, respectively.

### Replication analyses in SHIP-Trend

**Fig. 3:**
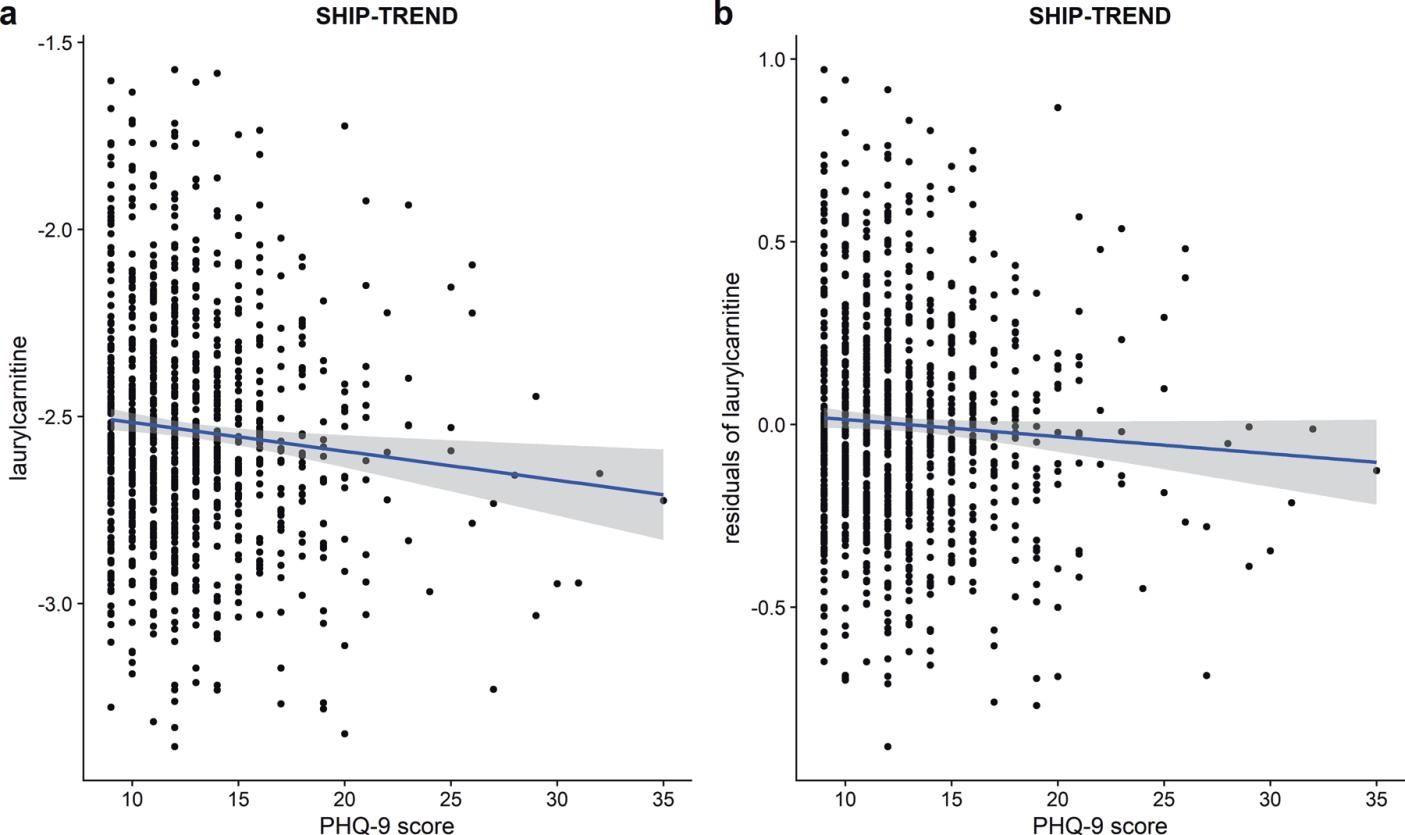
Scatterplots between the linear PHQ-9 score and laurylcarnitine values in the replication cohort SHIP-Trend. (**a**) displays the association between PHQ-9 scores on the *x*-axis and laurylcarnitine values on the *y*-axis, whereas (**b**) displays the association between PHQ-9 scores on the *x*-axis and the residuals of laurylcarnitine values regressed on the covariates age, sex, BMI, and the intake of antihypertensive drugs, antithrombotic agents, HMG CoA reductase inhibitors, nonsteroid anti-inflammatory and antirheumatic drugs as well as antidepressants on the *y*-axis. The blue lines represent linear regression fits.

Finally, we replicated the significant negative association between depression and laurylcarnitine in the SHIP-Trend cohort with a one-sided *p* value of 0.031 (*β* = −0.005, Fig. 3) for continuous PHQ-9 scores. Additional adjustment for thyroid gland hormone drugs (one-sided *p* value 0.031, *β* = −0.005) and no adjustment for antithrombotic agents and nonsteroid anti-inflammatory and antirheumatic agents (one-sided *p* value 0.027, *β* = −0.005) did not alter the results. In case of categorized depressive symptoms, we did find no significant associations between laurylcarnitine and depressive symptoms for neither a comparison between no vs. other/major (one-sided *p* value 0.084, *β* = −0.06), nor a comparison between no vs. major depressive symptoms (one-sided *p* value 0.327, *β* = −0.036), probably due to low case numbers. Nevertheless, the effect direction for the association between laurylcarnitine and depressive symptoms categories was replicated in SHIP-Trend.

## Discussion

The present study conducted a metabolome-wide association analysis comprising 353 unique serum metabolites in the general population sample of 1411 KORA F4 study participants. We were able to identify the organic compound laurylcarnitine to be significantly associated with depressive symptoms after thorough correction for multiple testing, and successfully replicated this finding in an independent general population sample of 968 SHIP-Trend study participants. Laurylcarnitine levels were significantly lower in individuals with other/major as well as in individuals with purely major depressive symptoms compared against individuals without depressive symptoms in KORA F4. Laurylcarnitine levels were also lower in serum specimens collected from individuals with other depressive symptoms, although without reaching statistical significance. This might point to a severity-dependent association between decreasing laurylcarnitine levels and increasing depressive symptoms.

Laurylcarnitine has not yet been identified as a major player in psychiatric disorders including depression, and its significant association with depressive symptoms in two independent general population cohorts comes as a surprise. Laurylcarnitine belongs to the chemical class of acylcarnitines [36], fatty acyl esters of the quaternary ammonium compound L-carnitine [37]. Acylcarnitines are involved in the transport of (long-chain) fatty acids from the cytosol across the mitochondrial membrane toward the mitochondrial matrix for subsequent β-oxidation [37]. Lower levels of acylcarnitines in depressed subjects might point to altered fatty acid oxidation and/or mitochondrial function in these individuals.

Aberrancies in mitochondrial energy production seem to contribute in important ways to the development of neuropsychiatric diseases, including depression [18, 38–43], post-traumatic stress disorder [44], schizophrenia [45], bipolar disorder [46], anxiety disorders [47], and psychosis [48]. Gardner et al. for instance, reported significant decreases in mitochondrial ATP production rates and mitochondrial enzyme ratios in MDD patients in comparison to healthy controls [39]. On the other hand, depressive disorders have been observed in patients with various mitochondrial disorders [49–55]. Interestingly, several antidepressants have been reported to influence neuronal mitochondrial function either positively or negatively [56]. In our study, we observed varying effect sizes for the association between laurylcarnitine and depressive symptoms in individuals using or not using antidepressants. However, case numbers in both subcohorts were rather small, and results should be interpreted with caution. Interestingly, a mice study reported altered brain energy metabolism and increased norepinephrine and serotonin content subsequent to chronic acetyl-L-carnitine intake [57], and acetyl-L-carnitine has been suggested for depression treatment due to its beneficial neuroplasticity effect, membrane modulation, and neurotransmitter regulation [58–60].

Several other studies reported distinct metabolic profiles of individuals with depressive disorders [8]. Liu et al. reported decreased plasma acylcarnitine levels, including laurylcarnitine and decenoyl carnitine, and other metabolites in MDD subjects [15]. Similar to our study, they discovered that metabolic differences between MDD patients and healthy controls increased with MDD severity. Another study investigated metabolic differences between hepatitis C-infected patients with and without pharmacologically induced immune-related depression [24], and found significantly lower levels of isoleucine in depressed patients, but no significant difference in acylcarnitines. In contrast to our study, Baranyi et al.’s study only included a small number of hepatitis C patients and investigated depression resulting from drug treatments [24]. Meanwhile, Setoyama et al. reported associations of 3-hydroxybutyrate, betaine, citrate, creatinine, and gamma-aminobutyrate with severity of depression independent from medication use as well as diagnostic differences [23]. Here, acetylcarnitine was a predictor for depression. In contrast to a recent finding evidencing acetylcarnitine deficiency as a key metabolic trait in MDD patients [61], our screening approach in KORA F4 failed to replicate this association (Supplementary Tables S3–5). Downregulated plasma acylcarnitines, namely propionylcarnitine, isobutyrylcarnitine, isovalerylcarnitine, and 2-methylbutyrylcarnitine, have also been reported in depressed subjects with and without HIV-infection [16]. MahmoudianDehkordi et al. recently conducted a targeted plasma metabolomics study of selective serotonin reuptake inhibitors (SSRIs) treatment in MDD patients and, interestingly, discovered increased short-chain and decreased medium and long-chain acylcarnitine levels, including laurylcarnitine, after 8 weeks of treatment [17]. The authors linked these alterations to SSRIs effects on β-oxidation and mitochondrial function. Interestingly, they additionally reported changes of several other lipids and amines, in particular methionine sulfoxide, serotonin, and sarcosine, in response to SSRI treatment. In contrast, we did not observe any alterations of serotonin in our KORA F4 screening approach (Supplementary Tables S3–5). Methionine sulfoxide and sarcosine were not included in our analyses. However, we observed a nominal downregulation of the amine kynurenine in subjects with other/major depressive symptoms (unadjusted *p* value 0.0194, Supplementary Table S3). Interestingly, the kynurenine pathway has already been identified as one of the possible key players in MDD-related metabolic alterations [62–64]. We also observed the nominal downregulation of additional lipids besides acylcarnitines in depressed individuals (Supplementary Tables S3–5) confirming findings of significant alterations in circulating lipid metabolites in a recent large-scale meta-analysis of depression [65].

Several limitations of our study have to be considered. Both KORA and SHIP-Trend predominantly include individuals of northern European ethnicity. Whether our findings can be generalizable to other ethnic groups would be the subject of further research. Secondly, no longitudinal metabolic and/or phenotypic data were available, preventing the investigation of metabolic changes in depressed individuals over time. Such investigations, especially in the context of acute depressive episodes, might enhance a deeper understanding of underlying biological mechanisms and potential causal relationships. Likewise, metabolic profiling of individuals with acute depressive episodes might further increase effect sizes and enable the identification of a broader metabolic depression profile. We diagnosed individuals with depressive symptoms present within the rather long period of 2 weeks. Thirdly, since depressive symptoms have only been assessed with the PHQ-9 questionnaire in both study cohorts, additional research considering other diagnostic instruments, e.g., the Hamilton Rating Scale for Depression or the Quick Inventory of Depressive Symptomatology, is warranted. Admittedly, despite the involvement of two large general population cohorts with a cross-over confirmation of the major findings, the empirical basis for causal conclusions remains relatively small, but provides a robust rationale to further explore these findings in clinical depression studies, e.g., the NESDA [66] or the recently initiated EMBARC study [67]. Such future investigations could increase effect sizes and share further insights into potential pharmacological mechanisms of laurylcarnitine and other metabolites in MDD patients, as, e.g., pointed out by [17, 22, 68–70].

Nevertheless, the strengths of our study are the broad coverage of the human serum metabolome, which facilitated an untargeted metabolome-wide association analysis to discover novel metabolic alterations in depression. Our findings were independent of the applied metabolomics measurement platform, as serum metabolites were assessed by Metabolon in KORA F4, but laurylcarnitine was measured with the Biocrates kit in SHIP-Trend. Overall good correlations, especially for acylcarnitines, between the two different platforms have been previously reported [71, 72], although metabolome coverage and measurement quality do differ between them. The successful replication of our finding based on the untargeted Metabolon approach in data measured by the targeted Biocrates kit underlines that the negative association between laurylcarnitine and depression was not driven by any analytical artefacts, but truly arises from biological variance. We thoroughly considered the potential influence of additional confounders, including age, sex, BMI, and drug intake, on our results. The replication of lower laurylcarnitine levels in depressed individuals was possible in an independent replication cohort. However, we were only able to report a replicated, statistically significant association for continuous PHQ-9 scores and not for depressive symptoms categories. Please note that our replication cohort SHIP-Trend was, in general, younger, had a lower BMI, and included more women than the discovery cohort KORA F4, which might contribute to the different impact of depressive symptoms on laurylcarnitine levels.

Nonetheless, this study presents a broad metabolome-wide association analysis including successful replication in probably two of the largest general population studies which assessed depressive symptoms with a harmonized instrument. The significantly lower levels of laurylcarnitine in depressed individuals might point towards a potential therapeutic target for the future development of depression treatments. Further large-scale metabolomics studies in populations of depressed patients with higher statistical power might facilitate additional insights into the underlying biological mechanisms of depression. Likewise, the determination of genetic contributions to altered acylcarnitine levels in depressed subjects might further enhance our understanding of underlying pathomechanisms including a possible role of mitochondrial dysfunction [42], as already similarly conducted for other metabolic depression markers [8, 22, 64, 73, 74].

## Supplementary information

Supplementary Material to A metabolome-wide association study in the general population reveals decreased levels of serum laurylcarnitine in people with depression.

Supplementary Table S3

Supplementary Table S4

Supplementary Table S5

Supplementary Table S7
