# Supplementary material for: A metabolome-wide association study in the general population reveals decreased levels of serum laurylcarnitine in people with depression
Source: Mol Psychiatry. 2021 Jun 4;26(12):7372–83. doi: 10.1038/s41380-021-01176-0 (PMC8873015; doi:10.1038/s41380-021-01176-0)
Supplement: Supplementary file 1 — Supplementary Material to A metabolome-wide association study in the general population reveals decreased levels of serum laurylcarnitine in people with depression. [file 41380_2021_1176_MOESM1_ESM.docx]

**Supplementary Material to** **A metabolome-wide association study in the general population reveals decreased levels of serum laurylcarnitine in people with depression.**

Helena U. Zacharias, Ph.D.^1,2,3^, Johannes Hertel, Ph.D.^1,4^, Hamimatunnisa Johar, Ph.D.^5,6^, Maik Pietzner, Ph.D.^7,8^, Karoline Lukaschek, P.D.^9^, Seryan Atasoy, Ph.D.^6,10^, Sonja Kunze, Ph.D.^5,11^, Henry Völzke, M.D.^12^, Matthias Nauck, M.D.^7,13^, Nele Friedrich, Ph.D.^7,13^, Gabi Kastenmüller, Ph.D.^14^, Hans J. Grabe, M.D.^1,15^, Christian Gieger, Ph.D.^5,11,16^, Jan Krumsiek, Ph.D.^14,17^, Karl-Heinz Ladwig Ph.D., M.D.^5,10,16^

*^1^Department of Psychiatry and Psychotherapy, University Medicine Greifswald, Greifswald, Germany; ^2^Department of Internal Medicine I, University Medical Center Schleswig-Holstein, Campus Kiel, Kiel, Germany; ^3^Institute of Clinical Molecular Biology, Kiel University and University Medical Center Schleswig-Holstein, Campus Kiel, Kiel, Germany; ^4^School of Medicine, National University of Ireland, Galway, Ireland; ^5^Institute of Epidemiology, Helmholtz Zentrum München, Neuherberg, Germany; ^6^Department of Psychosomatic Medicine and Psychotherapy, University of Gießen and Marburg, Gießen, Germany; ^7^Institute of Clinical Chemistry and Laboratory Medicine, University Medicine Greifswald, Greifswald, Germany; ^8^Computational Medicine, Berlin Institute of Health (BIH) at Charité – Universitätsmedizin Berlin, Germany; ^9^Institute of General Practice and Family Medicine, University Hospital of the Ludwig-Maximilians University of Munich, Munich, Germany; ^10^Department of Psychosomatic Medicine and Psychotherapy, Klinikum rechts der Isar, Technische Universität München, Munich, Germany; ^11^Research Unit of Molecular Epidemiology, Helmholtz Zentrum München, Neuherberg, Germany; ^12^Institute of Community Medicine, University Medicine Greifswald, Greifswald, Germany; ^13^German Center for Cardiovascular Research (DZHK e.V.), partner site Greifswald, Greifswald, Germany; ^14^Institute of Computational Biology, Helmholtz Zentrum München, Neuherberg, Germany; ^15^German Centre for Neurodegenerative Diseases (DZNE), Site Rostock/Greifswald, Germany; ^16^German Center for Diabetes Research (DZD e.V.), Neuherberg, Germany; ^17^Department of Physiology and Biophysics, Weill Cornell Medicine, Institute for Computational Biomedicine, Englander Institute for Precision Medicine, New York, USA.*

**Running title:** Reduced serum laurylcarnitine levels in depression

**Correspondence:**

Karl-Heinz Ladwig,

Department of Psychosomatic Medicine and Psychotherapy,

Klinikum rechts der Isar, Technische Universität München (TUM),

Langerstr. 3, 81675 Munich, Germany

E-mail: karl-heinz.ladwig@tum.de, ++49 89 41404311.

**Supplementary Materials and Methods**

*Psychological categorization in KORA F4*

‘Other’ or ‘major depressive symptoms’ were diagnosed if 2-4 or at least 5 of the PHQ-9 questions had been answered with at least “more than half of the days” in the past two weeks, and one of the diagnosed symptoms was depressed mood or anhedonia. Suicidal ideation was assessed in the KORA F4 study by asking the question “Have you had thoughts that you would be better off dead or of hurting yourself in some way in the last two weeks?”. Study participants who reported to suffer from these thoughts for several days, more than half of the days or nearly every day over the last two weeks were rated to suffer from suicidal ideation.

*Intake of antidepressants*

The use of antidepressants in the KORA F4 study was assessed with the IDOM-software based on information given by the study participants and the packages they brought to the personal interview. We considered tricyclic antidepressants (Anatomical Therapeutic Chemical (ATC)-code N06AA), selective serotonin reuptake inhibitors (ATC-code N06AB), mirtazapine antidepressants (ATC-code N06AG), lithium containing antidepressants (ATC-code N05AN01), as well as other antidepressants (ATC-code N06AX11) in our statistical analysis of the KORA F4 study.

In SHIP-Trend, the use of antidepressants within the last 7 days was assessed based on the packages brought to the interview by the study participants. For the statistical replication analysis, we considered selective serotonin reuptake inhibitors (ATC-code N06AB), non-selective monoamine reuptake inhibitors (ATC-code N06AA), St.-John’s-wort (ATC-code N06AP01), and other antidepressants (ATC-code N06AX).

*Cardiometabolic risk factors in KORA F4*

Standardized measurements of body height, weight, waist circumference, blood pressure, and serum lipids were performed during the physical examination as described previously (1). Diabetes mellitus was defined based on self-reported physician diagnosis or use of antidiabetic agents. Study participants without previously diagnosed diabetes underwent a standard 75-g oral glucose tolerance test after an overnight fasting of at least 8 hours. Study participants with known type-1 diabetes mellitus were excluded from statistical analysis. Antihypertensive medication was defined following the guidelines of the German Hypertension Society. In our statistical analysis in the KORA F4 cohort, we considered antihypertensive medication with beta-blockers, ACE inhibitors, angiotensin-antagonists or diuretics, lipid-lowering medication with statins, as well as antidiabetic and thyroid medication and systemic corticoid intake as potential confounders.

*Data preprocessing*

For statistical analyses in the KORA F4 cohort, 353 serum metabolites were included after excluding metabolites with more than 20% missing values. Missing values in the metabolite feature matrix were multivariately imputed by chained equations with predictive mean matching employing the *R* package “mice” (2). Gender was recoded into 0 and 1 for men and women, respectively. ‘No’, ‘other’, and ‘major depressive symptoms’ was either recoded into 0, 1, and 2, or 0 and 1 for ‘no’ and ‘other/major depressive symptoms’, respectively. The intake of drugs considered as confounders was recoded into 0 and 1 for taking or not taking a certain medication, respectively.

Metabolomics data pre-processing in SHIP-Trend included peak integration and concentration determination from calibration curves with Analyst software (Version 1.5.1, AB Sciex, Darmstadt, Germany). Afterwards, data were uploaded into Biocrates MetIDQ software, and metabolite concentrations were automatically calculated. More details are given in (3).

**Supplementary Tables**

Table S1. Confounder selection in KORA F4 and SHIP-Trend. The last column provides a rationale for the selection of the specific confounder, based on previous results from metabolome-wide association studies in KORA F4.

| **Confounder** | **KORA F4** | **SHIP-Trend** | **Rationale** |
| --- | --- | --- | --- |
| Demographic Parameters | Age, sex, BMI | Age, sex, BMI | significant metabolite-associations reported in (4) |
| Antihypertensive/  Cardiovascular drugs | antihypertensive medication with beta-blockers  ACE inhibitors  angiotensin-antagonists  diuretics  lipid-lowering medication with statins | antihypertensive drugs^1^  antihypertensive drugs^1^  antihypertensive drugs^1^  antihypertensive drugs^1^  HMG CoA reductase inhibitors | significant metabolite-associations reported in (5)  significant metabolite-associations reported in (5)  significant influence on metabolome reported in (6)  significant metabolite-associations reported in (5)  significant metabolite-associations reported in (5) |
| Antidiabetic drugs | antidiabetic drugs | -^2^ | significant influence on metabolome reported in (6) |
| Thyroid gland hormone drugs | thyroid gland hormone drugs | -^3^ | significant metabolite-associations with thyroid function reported in (7) |
| Corticoids | systemic corticoids | -^4^ | congruent with (8) |
| Antithrombotic drugs | -^5^ | antithrombotic drugs^5^ |  |
| Non-steroid anti-inflammatory and antirheumatic drugs | -^5^ | non-steroid anti-inflammatory and antirheumatic drugs^5^ |  |
| Antidepressants | tricyclic antidepressants  selective serotonin reuptake inhibitors | antidepressants^6^  antidepressants^6^ | congruent with (9)  congruent with (9) |

^1^SHIP-Trend summarizes all antihypertensive drugs in one variable. ^2^SHIP-Trend only includes non-diabetic patients. ^3^Initial analyses in SHIP-Trend did not include thyroid gland hormone drugs as confounders, however, we conducted additional sensitivity analyses with a modified drug confounder set in SHIP-Trend, confirming our original findings. ^4^In SHIP-Trend, only 8 study participants reported the intake of corticoids (compare to Supplementary Table S2). ^5^Antithrombotic and non-steroid anti-inflammatory and antirheumatic drugs, comprising, e.g., ibuprofen, had not been included in the initial screening approach in KORA F4, however, we conducted additional sensitivity analyses with a modified drug confounder set in SHIP-Trend, confirming our original findings. ^6^Due to the lower sample size in SHIP-Trend, antidepressants were summarized into one variable, comprising tricyclic antidepressants, selective serotonin reuptake inhibitors, St. John’s-wort, and other antidepressants (compare to Supplementary Table S2).

Table S2. Baseline characteristics of SHIP-Trend study participants (*n* = 968) stratified according to depression status. For continuous variables, values are expressed as mean ± standard deviation, for categorical variables, values are expressed as counts and frequencies.

|  | **no depressive symptoms** | **other/ major depressive symptoms** | **other depressive symptoms** | **major depressive symptoms** | ***p*-value no vs. other/ major depressive symptoms** | ***p*-value no vs. other depressive symptoms** | ***p*-value no vs. major depressive symptoms** |
| --- | --- | --- | --- | --- | --- | --- | --- |
| ***n* (%)** | 916 (95%) | 52 (5.4%) | 37 (3.8%) | 15 (1.6%) | - | - | - |
| **male (%)** | 404 (44%) | 22 (42%) | 15 (40.5%) | 7 (47%) | 0.912^a^ | 0.795^a^ | 1^a^ |
| **BMI [kg/m^2^]** | 27.32 ± 4.49 | 28.06 ± 6.17 | 28.13 ± 6.12 | 27.89 ± 6.52 | 0.397^b^ | 0.43^b^ | 0.74^b^ |
| **Age [years]** | 50.14 ± 13.72 | 47.94 ± 12.33 | 48.43 ± 13.26 | 46.73 ± 9.97 | 0.22^b^ | 0.449^b^ | 0.213^b^ |
| **Intake of antihypertensive drugs (%)** | 251 (27%) | 17 (33%) | 11 (30%) | 6 (40%) | 0.5^a^ | 0.902^a^ | 0.429^a^ |
| **Intake of antithrombotic agents (%)** | 62 (7%) | 5 (10%) | 2 (5%) | 3 (20%) | 0.613^a^ | 1^a^ | 0.138^a^ |
| **Intake of HMG CoA reductase inhibitors (%)** | 63 (7%) | 5 (10%) | 5 (14%) | 0 (0%) | 0.637^a^ | 0.226^a^ | 0.594^a^ |
| **Intake of anti-inflammatory and antirheumatic drugs, non-steroids (%)** | 73 (8%) | 8 (15%) | 7 (19%) | 1 (7%) | 0.105^a^ | 0.04^a^ | 1^a^ |
| **Intake of corticoids (%)** | 8 (1%) | 0 (0%) | 0 (0%) | 0 (0%) | 1^a^ | 1^a^ | 1^a^ |
| **Intake of thyroid gland hormone drugs (%)** | 86 (9%) | 7 (13%) | 5 (14%) | 2 (13%) | 0.4668^a^ | 0.5811^a^ | 0.942 |
| **Intake of antidepressants (%)** | 27 (3%) | 12 (23%) | 5 (14%) | 7 (47%) | 9.21e^-12a^ | 0.002^a^ | 1.458e^-16a^ |
| Intake of selective serotonin reuptake inhibitors (%) | 11 (1%) | 2 (4%) | 2 (5%) | 0 (0%) | 0.321^a^ | 0.15^a^ | 1^a^ |
| Intake of non-selective monoamine reuptake inhibitors (%) | 10 (1%) | 2 (4%) | 0 (0%) | 2 (13%) | 0.270^a^ | 1^a^ | 0.003^a^ |
| Intake of St.-John’s-wort (%) | 0 (0%) | 1 (2%) | 0 (0%) | 1 (7%) | 0.048^a^ | - | 0.0001^a^ |
| Intake of other antidepressants (%) | 8 (1%) | 9 (17%) | 3 (8%) | 6 (40%) | 1.81e^-16a^ | 0.001^a^ | 1.624e^-29a^ |

^a^*P*-values were calculated using Pearson’s Chi-squared test with Yates’ continuity correction. ^b^*P*-values were calculated using a two-sided *t*-test assuming unequal variance. Abbreviations: BMI, body mass index.

Table S3. Results of the screening for metabolic depression markers in KORA F4. Unadjusted, adjusted *p*-values according to Benjamini/Hochberg (BH), and relative differences (regression coefficients *β*) are given for the linear regression test comparing study participants without depression versus participants with other/major depressive symptoms combined. The first three columns provide the names of the metabolites or their Metabolon identifier, if not yet identified, and their corresponding sub- and superpathways as provided by Metabolon Inc.. The regression analysis included the confounders age, sex, body-mass index (BMI), and the intake of antihypertensive, cardiovascular, antidiabetic, and thyroid gland hormone drugs, as well as corticoids, and antidepressants.

Table S4. Results of the screening for metabolic depression markers in KORA F4. Unadjusted, adjusted *p*-values according to Benjamini/Hochberg (BH), and relative differences (regression coefficients *β*) are given for the linear regression test comparing study participants without depression versus participants with other depressive symptoms. The first three columns provide the names of the metabolites or their Metabolon identifier, if not yet identified, and their corresponding sub- and superpathways as provided by Metabolon Inc.. The regression analysis included the confounders age, sex, body-mass index (BMI), and the intake of antihypertensive, cardiovascular, antidiabetic, and thyroid gland hormone drugs, as well as corticoids, and antidepressants.

Table S5. Results of the screening for metabolic depression markers in KORA F4. Unadjusted, adjusted *p*-values according to Benjamini/Hochberg (BH), and relative differences (regression coefficients *β*) are given for the linear regression test comparing study participants without depression versus participants with major depressive symptoms. The first three columns provide the names of the metabolites or their Metabolon identifier, if not yet identified, and their corresponding sub- and superpathways as provided by Metabolon Inc.. The regression analysis included the confounders age, sex, body-mass index (BMI), and the intake of antihypertensive, cardiovascular, antidiabetic, and thyroid gland hormone drugs, as well as corticoids, and antidepressants.

Table S6. Carnitine metabolites measured and identified by the KORA F4 Metabolon approach and corresponding pseudonyms.

| **Metabolon name** | **Biocrates name^a^** | **IUPAC name** | **Other pseudonyms^b^** |
| --- | --- | --- | --- |
| carnitine | C0 | (3R)-3-hydroxy-4-(trimethylazaniumyl)butanoate | levocarnitine; 3-hydroxy-4-N-trimethylaminobutyrate; vitamin BT; L-gamma-trimethyl-beta-hydroxybutyrobetaine |
| 3-dehydrocarnitine |  | 3-oxo-4-(trimethylazaniumyl)butanoate |  |
| acetylcarnitine | C2 | (3R)-3-(acetyloxy)-4-(trimethylazaniumyl)butanoate | acetyl-L-carnitine; 3-(acetyloxy)-4-(trimethylammonio)butanoic acid |
| propionylcarnitine | C3 | (3R)-3-(propanoyloxy)-4-(trimethylazaniumyl)butanoate | O-propanoylcarnitine; (3R)-3-(Propionyloxy)-4-(trimethylammonio)butanoic acid |
| butyrylcarnitine | C4 | (3R)-3-(butanoyloxy)-4-(trimethylazaniumyl)butanoate | O-butanoylcarnitine; (3R)-3-(Butyryloxy)-4-(trimethylammonio)butanoic acid; butylcarnitine |
| 2-methylbutyroylcarnitine |  | 3-[(2-methylbutanoyl)oxy]-4-(trimethylazaniumyl)butanoate | methylbutyroyl-carnitine |
| isobutyrylcarnitine |  | (3R)-3-[(2-methylpropanoyl)oxy]-4-(trimethylazaniumyl)butanoate |  |
| succinylcarnitine |  | (3R)-3-[(3-carboxypropanoyl)oxy]-4-(trimethylazaniumyl)butanoate |  |
| isovalerylcarnitine | C5 | 3-[(3-methylbutanoyl)oxy]-4-(trimethylazaniumyl)butanoate | 3-methylbutyrylcarnitine |
| glutaroyl carnitine | C5-DC | (3R)-3-[(4-carboxybutanoyl)oxy]-4-(trimethylazaniumyl)butanoate | glutarylcarnitine |
| hydroxyisovaleroyl carnitine | C5-OH | (3R)-3-[(3-hydroxy-3-methylbutanoyl)oxy]-4-(trimethylazaniumyl)butanoate | 3-hydroxyisovalerylcarnitine |
| tiglyl carnitine^c^ | C5:1 | (3R)-3-{[(2E)-2-methylbut-2-enoyl]oxy}-4-(trimethylazaniumyl)butanoate | tiglylcarnitine |
| hexanoylcarnitine | C6 | (3R)-3-(hexanoyloxy)-4-(trimethylazaniumyl)butanoate | (R)-caproylcarnitine; L-carnitine hexanoyl ester; hexanoic acid |
| octanoylcarnitine | C8 | (3R)-3-(octanoyloxy)-4-(trimethylazaniumyl)butanoate | L-carnitine octanoyl ester |
| decanoylcarnitine | C10 | 3-(decanoyloxy)-4-(trimethylazaniumyl)butanoate | (+-)-decanoylcarnitine |
| cis-4-decenoyl carnitine |  | (3R)-3-[(4Z)-dec-4-enoyloxy]-4-(trimethylazaniumyl)butanoate |  |
| laurylcarnitine | C12 | (3R)-3-(dodecanoyloxy)-4-(trimethylazaniumyl)butanoate | lauroylcarnitine, dodecanoylcarnitine |
| 2-tetradecenoyl carnitine | C14:1 | (3R)-3-[(2E)-tetradec-2-enoyloxy]-4-(trimethylazaniumyl)butanoate | trans-2-tetradecenoyl-L-carnitine; |
| palmitoylcarnitine | C16 | (3R)-3-(hexadecanoyloxy)-4-(trimethylazaniumyl)butanoate | hexadecanoylcarnitine; L-carnitine palmitoyl ester |
| stearoylcarnitine | C18 | (3R)-3-(octadecanoyloxy)-4-(trimethylazaniumyl)butanoate | octadecanoylcarnitine |
| oleoylcarnitine | C18:1 | (3R)-3-[(9Z)-octadec-9-enoyloxy]-4-(trimethylazaniumyl)butanoate | octadecenoylcarnitine |

^a^Biocrates names are given according to the Biocrates AbsoluteIDQ p180 kit. ^b^Other pseudonyms are provided according to Biocrates AbsoluteIDQ p180 kit names inconsistent to the provided Metabolon name and/or according to the Human Metabolome Database (HMDB) (10). ^c^Tiglyl carnitine has been excluded from the screening approach in KORA F4 due to a high number of missing values.

Table S7. Results of the Pearson correlation analysis between acylcarnitines and all 353 metabolites included in the screening approach in KORA F4. For each pair-wise correlation, the Pearson’s correlation coefficient, as well as the unadjusted and according to Benjamini/Hochberg-adjusted *p*-values are given. Significant, i.e. adjusted *p*-value < 0.05, Pearson’s correlation coefficients are marked by an asterisk and strong correlation coefficients, i.e. absolute Pearson’s correlation coefficient > 0.5, are highlighted in yellow. Not yet identified metabolites are named by their Metabolon identifier.

**Supplementary Figures**

Figure S1. Volcanoplots illustrating the results of the screening for serum metabolic depression markers in KORA F4. (a) Comparison between study participants without depressive symptoms vs. study participants with other depressive symptoms. (b) Comparison between study participants without depressive symptoms vs. study participants with major depressive symptoms. The *x*-axis shows the log_2_-fold changes, whereas the *y*-axis shows the -log_10_ unadjusted *p*-values, respectively. Metabolites with Benjamini/Hochberg-adjusted two-sided *p*-values below the significance threshold of 0.05 are represented as triangles, whereas all other metabolites are represented as dots. All metabolites identified as carnitines are plotted in blue, whereas all other metabolites, including not yet identified metabolites, are plotted in red, respectively.

Figure S2. Estimated regression coefficients *β* (effect sizes) for the linear regression analyses between serum laurylcarnitine (outcome) and other or major depressive symptoms (predictors) stratified according to different subpopulations in KORA F4. The mean of the estimated coefficient is represented either as a triangle in the case of a significant association with laurylcarnitine (unadjusted two-sided *p*-value < 0.05), or as a dot in the case of a non-significant association with laurylcarnitine. The whiskers correspond to the 95% confidence intervals. Regression coefficients for a comparison between no vs. other depressive symptoms are plotted in red, and no vs. major depressive symptoms are plotted in orange, respectively. Dashed vertical lines represent regression coefficients in the complete cohort and *β* = 0 is marked by a solid black line. Abbreviations: antidepr. med., antidepressive medication; suic. id., suicidal ideation.

**References**

1. Rathmann W, Haastert B, Icks A, Löwel H, Meisinger C, Holle R, et al. High prevalence of undiagnosed diabetes mellitus in Southern Germany: Target populations for efficient screening. The KORA survey 2000. Diabetologia. 2003;46(2):182–9.

2. van Buuren S, Groothuis-Oudshoorn K. mice: Multivariate imputation by chained equations in R. J Stat Softw. 2011;45(3):1–67.

3. Masuch A, Pietzner M, Bahls M, Budde K, Kastenmüller G, Zylla S, et al. Metabolomic profiling implicates adiponectin as mediator of a favorable lipoprotein profile associated with NT-proBNP. Cardiovasc Diabetol [Internet]. 2018;17(1):1–12. Available from: https://doi.org/10.1186/s12933-018-0765-1

4. Krumsiek J, Mittelstrass K, Do KT, Stückler F, Ried J, Adamski J, et al. Gender-specific pathway differences in the human serum metabolome. Metabolomics. 2015;11(6):1815–33.

5. Altmaier E, Fobo G, Heier M, Thorand B, Meisinger C, Römisch-Margl W, et al. Metabolomics approach reveals effects of antihypertensives and lipid-lowering drugs on the human metabolism. Eur J Epidemiol. 2014;29(5):325–36.

6. Suhre K, Meisinger C, Döring A, Altmaier E, Belcredi P, Gieger C, et al. Metabolic Footprint of Diabetes: A Multiplatform Metabolomics Study in an Epidemiological Setting. PLoS One [Internet]. 2010 Nov 11;5(11):e13953. Available from: https://doi.org/10.1371/journal.pone.0013953

7. Pietzner M, Kacprowski T, Friedrich N. Empowering thyroid hormone research in human subjects using OMICs technologies. J Endocrinol. 2018;238(1):R13–29.

8. Vogt S, Wahl S, Kettunen J, Breitner S, Kastenmüller G, Gieger C, et al. Characterization of the metabolic profile associated with serum 25-hydroxyvitamin D: A cross-sectional analysis in population-based data. Int J Epidemiol. 2016;45(5):1469–81.

9. Altmaier E, Emeny RT, Krumsiek J, Lacruz ME, Lukaschek K, Häfner S, et al. Metabolomic profiles in individuals with negative affectivity and social inhibition: A population-based study of Type D personality. Psychoneuroendocrinology. 2013;38(8):1299–309.

10. Wishart DS, Feunang YD, Marcu A, Guo AC, Liang K, Vázquez-Fresno R, et al. HMDB 4.0: The human metabolome database for 2018. Nucleic Acids Res. 2018;46(D1):D608–17.
